# Association between Dietary Acid Load and Hyperuricemia in Chinese Adults: Analysis of the China Health and Nutrition Survey (2009)

**DOI:** 10.3390/nu15081806

**Published:** 2023-04-07

**Authors:** Min Zhang, Chen Ye, Ruoyu Wang, Zongfeng Zhang, Xiaojie Huang, Mairepaiti Halimulati, Meng Sun, Yuxin Ma, Zhaofeng Zhang

**Affiliations:** 1 Department of Nutrition & Food Hygiene, School of Public Health, Peking University Health Science Center, Haidian District, Beijing 100191, China; 2 National Institute for Nutrition and Health, Chinese Center for Disease Control and Prevention, Beijing 100050, China; 3 Beijing’s Key Laboratory of Food Safety Toxicology Research and Evaluation, Haidian District, Beijing 100191, China

**Keywords:** dietary acid load, hyperuricemia, potential renal acid load (PRAL), net endogenous acid production (NEAP), serum uric acid

## Abstract

Background: This study evaluates the association between dietary acid load (DAL) and hyperuricemia in Chinese adults. Methods: The China Health and Nutrition Survey (CHNS) in 2009 was used in this cross-sectional study. Potential renal acid load (PRAL) and net endogenous acid production (NEAP) were applied to estimate DAL. A multiple logistic regression model was used to test the relationship between DAL and hyperuricemia risk. Results: A total of 7947 participants were included in this study, of whom 1172 had hyperuricemia. The PRAL score was positively related to the prevalence of hyperuricemia, even when potential covariates were taken into account. In comparison with Q1, the ORs were 1.12 (95% CI, 0.92–1.38), 1.20 (95% CI, 0.97–1.47) and 1.42 (95% CI, 1.16–1.75) in Q2, Q3 and Q4. However, there was no significant relationship between NEAP scores and hyperuricemia. Every 10 g increase in energy-adjusted fat, protein and animal protein intakes caused a 10%, 17% and 18% increase in hyperuricemia risk, respectively (OR: 1.10, 95% CI: 1.04–1.16; OR: 1.17, 95% CI: 1.11–1.25; OR: 1.18, 95% CI: 1.12–1.24, respectively). An obvious linear correlation was also suggested by the restricted cubic spline. Conclusions: Hyperuricemia risk was associated with higher PRAL among Chinese adults. This means that a diet low in PRAL scores could be a very valuable uric acid-lowering dietary pattern.

## 1. Introduction

Hyperuricemia and gout have become serious worldwide public health issues owing to their high global prevalence, significant younger trend and huge impact on physical function, health-related quality of life and medical expenditures [1,2,3]. It has been reported that hyperuricemia is not only closely correlated with gout, but also with diverse comorbidities, including hypertension [4,5], coronary artery disease [6], heart failure [7], atrial fibrillation [8], chronic kidney disease [9,10], diabetes [11] and metabolic syndrome [12]. There are several studies indicating hyperuricemia may contribute to these diseases [13,14]. In addition, gout management remains poor, uric acid-lowering therapy is only prescribed to less than half of patients [15]. It is crucial to prevent hyperuricemia by targeting its risk factors. In regards to the etiology of hyperuricemia, apart from genetics, social factors and environmental conditions, dietary patterns may also contribute to the ongoing prevalence of hyperuricemia [16]. The Global Burden of Disease Analysis Report has identified intensive and detailed dietary management as a more effective intervention [17,18].

The most important factor affecting serum uric acid (SUA) levels is renal excretion, which accounts for approximately 90% of all cases of hyperuricemia [19]. The influencing factors of renal uric acid excretion include renal dysfunction, drug use, genetic defects and acid-base balance homeostasis [20,21]. Acid-base balance is greatly influenced by diet, which supplies acid or base precursors to the body [22]. By measuring dietary acid load (DAL), we could estimate acid-base changes in the organism due to food intake [23]. Some studies have indicated that a lower DAL can reduce SUA values and increase renal excretion of uric acid [24,25]. According to extensive Korean prospective cohort research, middle-aged and older adults with higher dietary acid load scores were more likely to have hyperuricemia [26]. A case-control study from China that included 290 subjects also concluded that an increased level of DAL was linked to an elevated hyperuricemia risk [27]. Thus, DAL has a crucial influence on SUA values, and it may be one of the main modifiable factors.

Two approaches are generally taken in assessing DAL, namely potential renal acid load (PRAL) and net endogenous acid production (NEAP) [22,28]. According to the intake of protein, phosphorus potassium, magnesium and calcium, one can calculate PRAL scores, whereas dietary protein and potassium are taken into account when calculating the NEAP score [29]. Low PRAL and NEAP scores indicate the alkali-forming potential of edible foods, while high levels indicate their acid-forming potential [26,30]. In general, DAL is low in fruits and vegetables, due to the abundance of potassium, which is exchanged with hydrogen ions to produce an alkalizing effect [31]. Some animal-source foods contain high amounts of methionine and phosphorus, which act as potential inorganic acid precursors, contributing to increased endogenous acid production and DAL [32].

Existing epidemiological evidence indicates that SUA values may be negatively affected by a high DAL, but prior studies have tended to focus on high-income countries. Several studies have demonstrated that the prevalence of hyperuricemia varies widely across dietary patterns, ethnic groups and geographic regions [16,33,34], which may lead to findings from foreign studies not necessarily applying to the Chinese population. On the relationship between DAL and hyperuricemia in China, only one case-control study including 290 subjects had been reported, which had a relatively smaller sample size and was not nationally representative. Therefore, there has been limited exploration of this issue in China, and more epidemiological evidence needs to be accumulated. Since hyperuricemia is prevalent in China, it is essential to examine the link between DAL and hyperuricemia, not only in light of current public health policies, but also to provide guidance on strategies to prevent and promote health problems in the future. To address these problems, the China Health and Nutrition Survey (CHNS) was applied to assess whether PRAL and NEAP scores were related to hyperuricemia among adults in China.

## 2. Materials and Methods

### 2.1. Data Collection and Subjects

This study analyzed data from the CHNS [35], a longitudinal prospective household survey involving 10 data collection surveys in multiple age groups, involving 7200 households with a total of 30,000 individuals in 15 provinces and municipal cities. Following the launch of the CHNS in 1989, follow-up surveys were carried out every two or three years. Information about biological sampling was gathered only in 2009. The data from the 2009 survey were utilized in our investigation.

**Figure 1:**
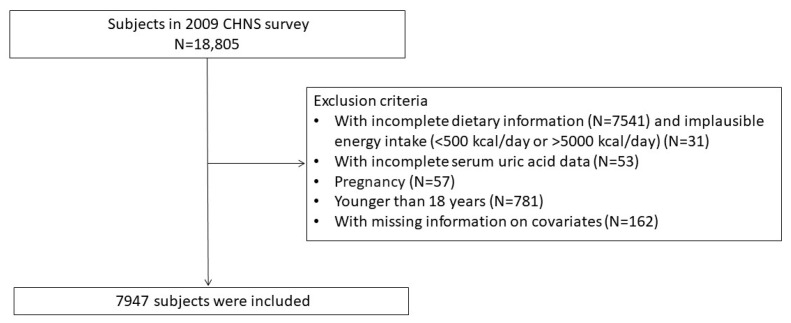
Flow chart of research participants selection.

A cross-sectional study was developed to collect the general demographics of Chinese inhabitants as well as their health and nutritional status. In 2009, 18,805 people took part in the survey. After excluding participants with incomplete dietary information (N = 7541), implausible energy intake (N = 31) (500 kcal/day or >5000 kcal/day), no serum uric acid data (N = 53) or missing data on covariates (N = 162), and those who were pregnant (N = 57) or younger than 18 years (N = 781), 7947 participants were eventually enrolled (Figure 1).

Before taking part in the study, each individual provided informed consent. The Carolina Population Center at the University of North Carolina at Chapel Hill and the National Institute for Nutrition and Health at the Chinese Center for Disease Control and Prevention (CCDC) both gave their approval to the investigation (2015017).

### 2.2. Dietary Data, PRAL and NEAP Scores

Personal dietary consumption information was obtained by asking participants to record their daily food varieties and intake over 24 h periods. The dietary records needed to be investigated for 2 weekdays and 1 day at the weekend. Meanwhile, three days’ worth of food weighing was used to obtain information about edible oil and condiments. The questionnaire used to collect food consumption in this study was the validated Food Frequency Questionnaire (FFQ) [36]. Daily energy and dietary nutrients were calculated from China’s Food Composition Tables (2002 and 2004).

We used the following equation to calculate PRAL and NEAP scores [28]

PRAL (mEq/d) = 0.4888 × protein intake (g/d) + 0.0366 × phosphorus (mg/d) − 0.0205 × potassium (mg/d) − 0.0125 × calcium (mg/d) − 0.0263 × magnesium (mg/d);

NEAP (mEq/d) = (54.5 × protein intake (g/d) ÷ potassium intake (mEq/d)) − 10.2.

### 2.3. Definition of Hyperuricemia

In the present study, hyperuricemia was diagnosed in the light of the definition of hyperuricemia and gouty conditions, and Chinese expert consensus on the treatment of hyperuricemia and gout [37,38]. For men, SUA ≥ 420 µmol/L (7 mg/dL) can be diagnosed as hyperuricemia, with a level of SUA ≥ 360 µmol/L (6 mg/dL) for women.

### 2.4. Other Variables

Information about an individual’s gender, age, residence area, marital status, education level, life habits, anthropometric indicators and medical histories was obtained via the CHNS questionnaire. The area of residence included both rural and urban areas. There were six grades of education: none, elementary, middle, high school, technical school, and university or college. Marital status was classified into three categories: single, married and other. Life habits included alcohol intake and smoking status, and required yes or no answers. Body mass index (BMI) was classified into four categories: less than 18.5 was considered low weight,18.5–23.9 normal, 24 to 27.9 overweight and 28 or more obese. Medical histories included diabetes (yes/no) and hypertension (yes/no). Based on serum creatinine, we calculated the estimated glomerular filtration rate (eGFR) according to the chronic kidney disease–Epidemiology Collaboration (CKD-EPI) equation [39].

### 2.5. Statistical Analysis

In our descriptive analysis, categorical variables were calculated by chi-squared tests, and the results were expressed as counts (percentages). Student’s *t*-test (normal) and the Wilcoxon rank sum test (non-normal) were employed to calculate the differences in the continuous variables, and the results were expressed as means (standard deviations, SDs) and medians (interquartile ranges, IQRs). The final covariates included in the study were judged according to whether the basic characteristics of the two groups were different.

We adopted a multilevel logistic regression model to analyze whether hyperuricemia and DAL (PRAL and NEAP) were related. Four groups were identified based on PRAL and NEAP scores quartiles. The odds ratio (OR) was calculated based on the quartiles of the DAL score. The reference group was Q1, the lowest group. Considering that dietary fiber and water intake can affect the excretion of uric acid, we adjusted for them as covariates. Three models were developed: model 1 without covariate adjustment; model 2 adjusted for age, gender, residence area, smoking status, marital status, alcohol intake, education level, BMI, hypertension, diabetes and eGFR; and model 3 further adjusted for dietary fiber and water intake. Analysis of subgroups was conducted by gender and age (<60 and ≥60 years).

In order to assess whether dietary fiber intake (5.7 g/1000 Kcal; >5.7 g/1000 Kcal) and water intake (<1500 mL (female), <1700 (male); ≧1500 mL (female), ≧1700 (male)) could modify the association between DAL and hyperuricemia, we conducted interaction analyses.

We examined linearity using multivariable-adjusted restricted cubic splines (model 3) and investigated the dose–response correlation between DAL and the prevalence of hyperuricemia.

SPSS Statistics 23 was used for all the analyses. The figures were produced in R version 4.2.2 and Graphpad Prism 9.4.1. *p* < 0.05 was defined as statistically significant.

## 3. Results

### 3.1. Participants Characteristics

This study included 7947 subjects. The participants’ mean age was 50.7 ± 15.0 years, of whom 4215 (53%) were females, 3732 (47%) were males, and 6701 (84.3%) were married. The rural population was 5520 (69.5%) and the urban population was 2427 (30.5%). The history of alcohol consumption and smoking was 1652 (20.8%) and 2468 (31.1%), respectively. In total, 2531 subjects had hypertension (31.8%) and 606 subjects had diabetes (7.6%).

A total of 1172 subjects in our study had hyperuricemia, a prevalence of 14.7% (19% for men and 11% for women). The median values for PRAL and NEAP were 21.9 mEq/d and 75.2 mEq/d, respectively. The distribution of subject characteristics is summarized in Table 1.

The subjects’ total energy, water intake and energy-adjusted carbohydrate, protein, fat, cholesterol, dietary fiber and mineral intake are shown in Table 2. In comparison with the non-hyperuricemia participants, those with hyperuricemia consumed more total energy, energy-adjusted protein, animal protein (excluding milk and eggs), fat, calcium and phosphorus, while the intake of energy-adjusted carbohydrate, dietary fiber and plant protein was dramatically lower. There was no discrepancy the groups regarding the intake of potassium and magnesium.

### 3.2. Association between DAL and Hyperuricemia

#### 3.2.1. PRAL and Hyperuricemia

In all three logistic regression models, an increased prevalence of hyperuricemia and elevated PRAL scores were related (Table S1). In comparison with Q1, the model 1 ORs were 1.28 (95% CI, 1.06–1.55), 1.44 (95% CI, 1.20–1.74) and 1.88 (95% CI, 1.57–2.24) in Q2, Q3 and Q4. After adjusting for model 2 and model 3, the positively significant association remained, shown in Table 3. When PRAL was treated as a continuous covariate, there was also a strikingly positive correlation (results are not displayed).

#### 3.2.2. NEAP and Hyperuricemia

As shown in models 1 and 2, the higher the NEAP scores, the greater the hyperuricemia risk. The ORs in model 2 were 1.25 (95% CI, 1.03–1.53), 1.30 (95% CI, 1.07–1.58) and 1.40 (95% CI, 1.15–1.70) in Q2, Q3 and Q4. However, confounding factors were further adjusted with model 3 (model 3), where NEAP scores and hyperuricemia risk did not appear to be correlated (*p*-values > 0.05), shown in Table 4. The complete results are reported in Table S2. In multiple logistic regression, both still lack correlation when NEAP was performed as a continuous variable (the results are not displayed).

#### 3.2.3. Association between DAL and Hyperuricemia after Gender and Age Stratification

**Figure 2:**
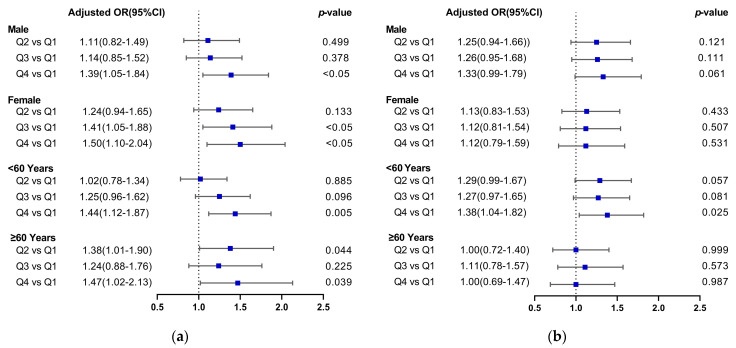
Forest plots of the relationship between dietary acid load (DAL) and the risk of hyperuricemia. (Plot (**a**) PRAL and hyperuricemia risk; Plot (**b**) NEAP and hyperuricemia risk).

The results of PRAL stratified by gender showed that in the male group, Q4 participants were more likely than Q1 participants to suffer from hyperuricemia after controlling for all confounding variables (Q4: OR 1.39, 95% CI 1.05–1.84). When all the confounders were taken into account, Q3 and Q4 female subjects also demonstrated an increased risk of hyperuricemia versus Q1 female subjects (Q3: OR 1.41, 95% CI 1.08–1.88; Q4: OR 1.50, 95% CI 1.10–2.04). After controlling for all potentially confounding factors, the age-stratified results indicated a link between PRAL and hyperuricemia prevalence. In the age <60 years group, compared with Q1, the OR for Q4 was 1.44 (95% CI, 1.12–1.87). For the age ≥60 years subjects, in comparison with Q1, the ORs were 1.38 (95% CI, 1.01–1.90), 1.24 (95% CI, 0.88–1.76) and 1.47 (95% CI, 1.02–2.13) in Q2, Q3 and Q4. The results are shown in Figure 2a.

According to subgroup analysis, elevated NEAP was significantly associated with hyperuricemia among the age <60 years group. After adjusting for all confounders, compared with Q1, the OR for Q4 was 1.38 (95% CI, 1.04–1.82). Concerning the gender groups and age ≥60 years group, the NEAP and hyperuricemia risks did not appear to be related after all the confounding factors were taken into account, as shown in Figure 2b.

### 3.3. Restricted Cubic Spline (RCS) Analysis of DAL and Risk of Hyperuricemia

**Figure 3:**
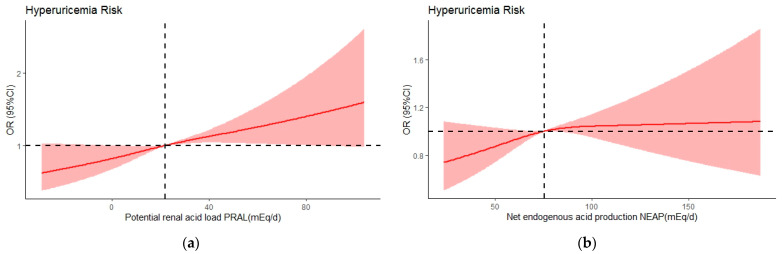
Multivariate adjusted odds ratios (ORs) by PRAL (**a**) and NEAP (**b**) for risk of hyperuricemia, in model 3. A knot is located at the 10th, 50th and 90th percentiles. The median intakes were set as references (gray dashed lines) (OR = 1.00). CI, confidence interval.

Multivariable-adjusted restricted cubic spline analyses showed a significant linear relationship between PRAL and hyperuricemia risk (*p* for overall <0.005 and *p* for nonlinearity >0.05), as displayed in Figure 3a. However, it was identified that NEAP and hyperuricemia were not correlated (*p* for overall = 0.2137), shown in Figure 3b. Meanwhile, when PRAL exceeded 22 mEq/day, the OR increased significantly (Figure 3).

### 3.4. Association between Dietary Intakes and Hyperuricemia

**Figure 4:**
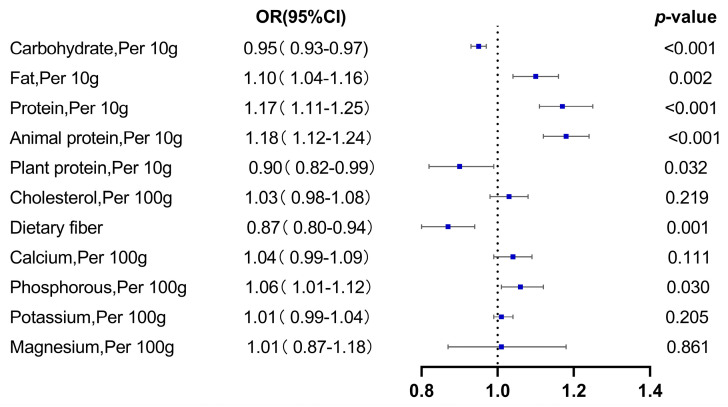
Dietary intake and hyperuricemia risk.

After adjustment, every 10 g increase in energy-adjusted fat, protein and animal protein intakes caused a 10%, 17% and 18% increase in hyperuricemia risk, respectively (OR: 1.10, 95% CI: 1.04–1.16; OR: 1.17, 95% CI: 1.11–1.25; OR: 1.18, 95% CI: 1.12–1.24, respectively). For each 100 g of energy-adjusted phosphorus consumed, the hyperuricemia risk increased by 6% (OR: 1.06, 95% CI: 1.01–1.12). Intake of energy-adjusted carbohydrate, plant-derived proteins and dietary fiber may reduce the risk of hyperuricemia (OR: 0.95, 95% CI: 0.93–0.97; OR: 0.90, 95% CI: 0.82–0.99; OR: 0.87, 95% CI: 0.80–0.94, respectively). There was no correlation between energy-regulated potassium, cholesterol, calcium and magnesium intake and the risk of hyperuricemia (Figure 4).

### 3.5. Interaction Analysis

From Table 2, we observed that dietary fiber and water intake were distinct in the two groups, so interaction analysis was performed. No interaction was found between dietary fiber and water intake and DAL, as regards the effect on serum uric acid. Neither additive nor multiplicative interactions were observed (Tables S3–S6).

## 4. Discussion

This study is the first investigation in China to examine the relationship between DAL and hyperuricemia in a large and geographically representative sample size, to the best of the authors’ knowledge. Our analysis revealed that the PRAL score was linked to the prevalence of hyperuricemia, following a linear correlation. The NEAP score and the hyperuricemia risk were positively related in the age <60 years group, while no significant results were found in the overall participants, male, female and ≥60 years groups. Even when confounding factors were taken into account, this correlation still existed. Additionally, we identified that the intake of energy-adjusted protein, fat, animal protein and phosphorus was positively linked to the risk of hyperuricemia, while energy-adjusted dietary fiber, plant protein and carbohydrate were inversely correlated.

In other countries, the association between DAL and hyperuricemia had been examined. An analysis of 6894 German adults showed that PRAL and SUA values had a positive correlation, and in the highest PRAL score group, the hyperuricemia risk was higher than that in the lowest group (OR: 0.60; 95% CI: 0.43, 0.83) [40], which is consistent with our discovery. Another study of the same sample from Germany showed that elevated NEAP was related to higher SUA levels [41]. Nevertheless, we found a lack of significance in the correlation between NEAP scores and hyperuricemia risk among the total participants. This difference may be explained by the estimation formulas of PRAL and NEAP. PRAL includes dietary protein intake and numerous minerals such as calcium, magnesium, potassium and phosphorus, whereas NEAP considers only dietary protein intake and potassium, assuming that mineral cations other than K are negligible, which may affect accuracy. In addition, the varying findings may also be attributable to differences in study design and the inclusion criteria for subjects, or due to differences in diet quality, genetics, lifestyle and sociodemographic characteristics. The underlying mechanism of the influence between DAL and uric acid has not been fully determined. A crossover intervention study from Japan showed that consumption of an alkaline diet was more beneficial for uric acid excretion than a more acidic diet, suggesting that an acidic environment may enhance reabsorption capacity [25]. Other studies have also shown that DAL affects the complicated mechanism of SUA excretion and reabsorption in the proximal tubule by altering urinary pH [42]. Consistently, inadequate renal uric acid excretion has been considered to be the major mechanism for hyperuricemia in most people [19]. Low PRAL is beneficial in promoting renal uric acid excretion and lowering SUA, so it may represent a potential urate-lowering dietary pattern.

We further explored the risk of hyperuricemia based on nutrient intakes, and found that a higher energy-adjusted fat and protein intake was linked to hyperuricemia prevalence in Chinese adults. The finding is consistent with previous results, which have suggested that Western dietary patterns with high protein and fat content can directly contribute to the modern hyperuricemia epidemic [43]. We also revealed that energy-adjusted animal protein intake increased the risk of hyperuricemia, while plant protein intake showed the opposite relationship. In general, it is thought that protein and hyperuricemia risk are often influenced by purines. There is evidence that consuming an excessive amount of animal protein (excluding milk and eggs) is accompanied by an excessive intake of purines, which can affect serum uric acid levels [44,45,46]. Moreover, animal-derived proteins are rich in xanthine and hypoxanthine, which can be directly metabolized to uric acid and have a large impact on serum uric acid values, while for plant-derived protein, including all cereals, legumes and soy products, more than 60% of purines are in the form of adenine and guanine, which have little impact on uric acid [47]. From the perspective of DAL, animal proteins contain high amounts of phosphorus, which can elevate PRAL, whereas plant proteins contain phosphorus as phytic acid, which is less bioavailable and has a diminished acidification effect. In addition, glutamate in plant proteins may have a neutral effect on acid loading [48]. In combination with these results, we suggest that the current approach to uric acid-lowering dietary therapy with a low purine diet as the primary intervention needs to be reconsidered. Concerning dietary recommendations on uric acid-lowering, reducing animal-derived protein intake, but not limiting plant-derived protein, could be considered to sustain the acid-base balance of the organism.

Our study found that hyperuricemic subjects consumed less energy-adjusted dietary fiber than non-hyperuricemic subjects. In addition, the results of multiple logistic regressions indicated a reduced risk of hyperuricemia with high dietary fiber ingestion. This association persisted while adjusting for full confounding factors. The positive effect of dietary fiber ingestion on reducing hyperuricemia risk had been mentioned several times. A study from Korea comparing the nutritional intake and dietary quality of hyperuricemia and non-hyperuricemia groups demonstrated that subjects with hyperuricemia had a lower dietary fiber intake than the non-hyperuricemic subjects [49]. A study analyzing NHANES data indicated that adults who consumed lower dietary fiber had a greater risk of hyperuricemia [50]. For the prevention and treatment of hyperuricemia, we wish to emphasize that increasing dietary fiber intake is also one of the most effective interventions. It is necessary to develop and implement effective health education programs to increase public awareness of the benefits of dietary fiber.

A correlation between the risk of hyperuricemia and energy-regulated calcium, potassium and magnesium intake was not observed. It is generally believed that calcium, potassium and magnesium have a high alkalinizing capacity in favor of lower PRAL [29]. There are a few possible explanations for this discrepancy. One study from Rotterdam points out that an important contributor to DAL is protein intake [51]. It should be borne in mind that the food and nutrients consumed are not a single nutrient in action. When identifying the acidifying or alkalizing effects of foods, it is more important to consider the overall diet. Moreover, it is worth considering that patients with hyperuricemia may change their diet to consciously increase some foods that are full of nutrients such as calcium, potassium and magnesium. Consequently, the association between DAL and hyperuricemia may be bidirectional. Overall, the definite reasons for these observations are not yet clear, and more studies are essential to verify the relationships.

Our research has several strengths. First, a large and nationally representative sample was used to evaluate whether there was an existing relationship between DAL and the prevalence of hyperuricemia. Therefore, the results are reliable and typical. Second, when a broad range of confounding variables (dietary and non-dietary) were taken into account, the positive association of PRAL with hyperuricemia remained significant. Our findings not only accumulate epidemiological evidence for exploring the association between DAL and hyperuricemia in Chinese adults, but also demonstrate that a diet low in PRAL scores is likely to be an effective dietary management strategy to prevent elevated uric acid levels.

Our study also had some limitations. Firstly, DAL and hyperuricemia were assessed cross-sectionally, which limits measuring their causal association. Future studies could be carried out prospectively and longitudinally to verify these findings. Secondly, the ascertainment of hyperuricemia was based on only one fasting serum uric acid value of the subjects; however, fasting serum uric acid levels need to be measured on two different days, according to the guidelines for diagnosing hyperuricemia. In addition, there was no medically documented information on the use of anti-gout medications, hyperuricemia and gout history in the CHNS questionnaire. Therefore, SUA levels may not reflect the actual situation of individuals. Thirdly, although we used validated measures (PRAL and NEAP) to evaluate the overall DAL, the calculation based on dietary intake is an indirect method rather than a direct measurement. Due to individual factors, nutrient absorption in the gastrointestinal tract may vary considerably, but this is not considered by the calculated equations [52]. Finally, due to objective conditions, we did not consider the influence of genetic factors. Future research could clarify the influence of genetic polymorphisms and nutritional factors on hyperuricemia, which may provide individuals with more accurate uric acid-lowering treatments.

## 5. Conclusions

In summary, elevated PRAL was correlated with hyperuricemia risk among Chinese adults. A significant linear correlation was also suggested by the multivariable-adjusted restricted cubic spline. This means that a low PRAL diet has the potential to lower uric acid and could be a very valuable uric acid-lowering dietary pattern. However, no significant results were seen for the relationship between NEAP scores and hyperuricemia. Further research to verify this association is warranted.

## Figures and Tables

**Table 1:** Subject characteristics based on hyperuricemia status.

Variable	Overall (*n* = 7497)	Hyperuricemia (*n* = 1172)	Non-Hyperuricemia (*n* = 6775)	*p*-Value
Age (years)	50.7 (15.0)	53.4 (15.1)	50.2 (14.9)	<0.0001
Gender				
Male	3732 (47%)	708 (60.4%)	3024 (44.6%)	<0.0001
Female	4215 (53%)	464 (39.6%)	3751 (55.4%)	
PRAL ^1^ (mEq/d)	21.9 (20.3)	25.3 (22)	21.5 (19.9)	<0.0001
NEAP ^2^ (mEq/d)	75.2 (28.9)	78.8 (26.6)	74.5 (29.1)	<0.0001
Marital status				
Single	464 (5.8%)	58 (4.9%)	406 (6.0%)	<0.05
Married	6701 (84.3%)	970 (82.8%)	5731 (84.6%)	
Other	782 (9.8%)	144 (12.3%)	638 (9.4%)	
Region				
Urban	2427 (30.5%)	416 (35.5%)	2011 (29.7%)	<0.0001
Rural	5520 (69.5%)	756 (64.5%)	4764 (70.3%)	
Education level				
None	1932 (24.3%)	289 (24.7%)	1643 (24.3%)	<0.05
Elementary school	1584 (19.9%)	220 (8.8%)	1364 (20.1%)	
Middle school	2619 (33.0%)	355 (30.3%)	2264 (33.4%)	
High school	914 (11.5%)	140 (11.9%)	774 (11.4%)	
Technical or vocational school	538 (6.8%)	98 (8.4%)	440 (6.5%)	
University or college	360 (4.5%)	70 (6.0%)	290 (4.3%)	
Smoking status				
No	5479 (68.9%)	733 (62.5%)	4746 (70.1%)	<0.0001
Yes	2468 (31.1%)	439 (37.5%)	2029 (29.9%)	
Alcohol intake				
No	6295 (79.2%)	823 (70.2%)	5472 (80.8%)	<0.0001
Yes	1652 (20.8%)	349 (29.8%)	1303 (19.2%)	
Hypertension				
Yes	1048 (13.2%)	27(33.3%)	775 (11.4%)	<0.0001
No	6899 (86.8%)	899 (76.7%)	6000 (88.6%)	
Diabetes				
Yes	227 (2.9%)	56 (4.8%)	171 (2.5%)	<0.0001
No	7720 (97.1%)	1116 (95.2%)	6604 (97.5%)	
BMI ^3^ (kg/m^2^)				
<18.5	490 (6.2%)	33 (2.8%)	457 (6.7%)	<0.0001
18.5–23.9	4260 (53.6%)	456 (38.9%)	3804 (56.1%)	
24–27.9	2323 (30.5%)	474 (40.4%)	1949 (28.8%)	
≥28	774 (9.7%)	209 (17.8%)	565 (8.3%)	
eGFR ^4^ (mL/min/1.73 m^2^)	79.2 (16.6)	71.1 (18.6)	80.6 (16.0)	<0.0001

^1^ PRAL: potential renal acid load; ^2^ NEAP: net endogenous acid production; ^3^ BMI: body mass index; ^4^ eGFR: estimated glomerular filtration rate.

**Table 2:** Energy and nutrient intakes (energy-adjusted, per 1000 kcal).

Nutrients	Hyperuricemia	Non-Hyperuricemia	*p*
Energy (Kcal)	1982.93 (1518.35–2395.97)	1863.96 (1492.45–2308.99)	<0.0001
Carbohydrate (g/1000 kal)	158.50 (136.12–181.42)	167.13 (146.36–188.99)	<0.0001
Protein (g/1000 kal)	41.01 (35.01–50.05)	38.22 (32.96–45.92)	<0.0001
Animal protein (g/1000 kal)	18.17 (9.34–27.78)	13.44 (6.30–23.30)	<0.0001
Plant protein (g/1000 kal)	21.27 (17.44–26.16)	22.62 (18.63–27.43)	<0.0001
Fat (g/1000 kal)	24.39 (16.06–31.86)	21.84 (13.82–29.41)	<0.0001
Cholesterol (mg/1000 kal)	186.25 (101.74–284.38)	165.24 (82.39–266.81)	<0.0001
Dietary fiber (g/1000 kal)	5.31 (4.11–6.98)	5.78 (4.36–7.68)	<0.0001
Calcium (mg/1000 kal)	216.29 (164.78–298.15)	206.77 (156.40–277.69)	<0.0001
Phosphorous (mg/1000 kal)	568.92 (499.88–657.39)	559.92 (493.77–636.72)	0.002
Potassium (mg/1000 kal)	996.04 (833.09–1224.47)	993.06 (829.27–1208.59)	0.321
Magnesium (mg/1000 kal)	159.41 (136.44–186.82)	161.59 (139.77–187.17)	0.112
Water intake	960.00 (720.00–1440.00)	960.00 (720.00–1334.40)	<0.0001

**Table 3:** Odds ratios (ORs) and 95% confidence intervals (CIs) for hyperuricemia risk based on PRAL.

PRAL	Model 1		Model 2		Model 3	
	OR (95% CI)	*p*	OR (95% CI)	*p*	OR (95% CI)	*p*
Q1	1 (ref)		1 (ref)		1 (ref)	
Q2	1.28 (1.06–1.55)	0.011	1.18 (0.96–1.44)	0.114	1.12 (0.92–1.38)	0.259
Q3	1.44 (1.20–1.74)	<0.01	1.27 (1.04–1.56)	0.017	1.20 (0.97–1.47)	0.089
Q4	1.88 (1.57–2.24)	<0.01	1.53 (1.26–1.86)	<0.01	1.42 (1.16–1.75)	<0.01

**Table 4:** Odds ratios (ORs) and 95% confidence intervals (CIs) for hyperuricemia risk based on NEAP.

NEAP	Model 1		Model 2		Model 3	
	OR (95% CI)	*p*	OR (95% CI)	*p*	OR (95% CI)	*p*
Q1	1 (ref)	0.001	1 (ref)		1 (ref)	
Q2	1.39 (1.15–1.67)	<0.001	1.25 (1.03–1.53)	<0.05	1.19 (0.97–1.45)	0.104
Q3	1.54 (1.28–1.86)	<0.001	1.30 (1.07–1.58)	<0.01	1.19 (0.97–1.47)	0.100
Q4	1.69 (1.40–2.02)	<0.001	1.40 (1.15–1.70)	<0.01	1.25 (1.00–1.56)	0.052

## Data Availability

Publicly available datasets were analyzed in this study. This data can be found here: https://www.cpc.unc.edu/projects/china/data (accessed on 10 December 2022).

## Supplementary Materials

The following supporting information can be downloaded at: https://www.mdpi.com/article/10.3390/nu15081806/s1, Table S1: Odds ratios (OR) and 95% confidence intervals for hyperuricemia by PRAL; Table S2: Odds ratios (OR) and 95% confidence intervals for hyperuricemia by NEAP; Table S3: Outcome of multiplicative interaction (PRAL); Table S4: Outcome of multiplicative interaction (NEAP); Table S5: Outcome of additive interaction (PRAL); Table S6: Outcome of additive interaction (NEAP).
