# Supplementary material for: Association between Dietary Acid Load and Hyperuricemia in Chinese Adults: Analysis of the China Health and Nutrition Survey (2009)
Source: Nutrients. 2023 Apr 7;15(8):1806. doi: 10.3390/nu15081806 (PMC10144330; doi:10.3390/nu15081806)
Supplement: Supplementary file 1 [file nutrients-15-01806-s001.zip › nutrients-2288897-supplementary.pdf]

Supplementary Materials

Table S1: Odds ratios (OR) and 95% confidence intervals for hyperuricemia by PRAL.

	**Crude model**	**P**	**model 2**	**P**	**model 3**	**P**
Age			0.97(0.97-0.98)	<0.01	0.97(0.97-0.98)	<0.01
Gender (Female: Male)			0.51(0.43-0.59)	<0.01	0.51(0.44-0.59)	<0.01
PRAL						
Q1	1(ref)		1(ref)		1(ref)	
Q2	1.28(1.06-1.55)	0.011	1.18(0.96-1.44)	0.114	1.12(0.92-1.38)	0.259
Q3	1.44(1.20-1.74)	<0.01	1.27(1.04-1.56)	0.017	1.20(0.97-1.47)	0.089
Q4	1.88(1.57-2.24)	<0.01	1.53(1.26-1.86)	<0.01	1.42(1.16-1.75)	<0.01
Marital status						
Married: Single			0.78(0.56-1.09)	0.145	0.78(0.56-1.09)	0.145
Other: Single			1.02(0.68-1.54)	0.929	1.01(0.67-1.52)	0.964
Region (Rural: Urban)			0.85(0.74-0.98)	0.026	0.85(0.74-0.98)	0.023
Education level						
Elementary school: None			0.90(0.73-1.11)	0.328	0.90(0.73-1.11)	0.322
Middle school: None			0.90(0.73-1.10)	0.286	0.90(0.73-1.10)	0.294
High school: None			0.96(0.74-1.24)	0.763	0.97(0.75-1.25)	0.805
Technical or vocational school: None			1.18(0.88-1.58)	0.259	1.19(0.89-1.60)	0.239
University or college: None			1.11(0.79-1.56)	0.549	1.13(0.80-1.59)	0.486
Smoking status (Yes: No)			0.96(0.80-1.14)	0.61	0.96(0.81-1.14)	0.638
Alcohol intake (Yes: No)			1.52(1.29-1.80)	<0.01	1.53(1.29-1.81)	<0.01
Hypertension (Yes: No)			1.43(1.23-1.65)	<0.01	1.43(1.24-1.66)	<0.01
Diabetes (Yes: No)			1.08(0.87-1.36)	0.482	1.09(0.87-1.36)	0.477
eGFR（ml/min/1.73m2）			0.95(0.94-0.95)	<0.01	0.95(0.94-0.95)	<0.01
BMI (kg/m2)						
<18.5: 18.5-24			0.58(0.40-0.85)	<0.01	0.57(0.39-0.84)	<0.01
24-28: 18.5-24			1.95(1.68-2.26)	<0.01	1.97(1.70-2.29)	<0.01
⩾28: 18.5-24			3.07(2.50-3.75)	<0.01	3.09(2.53-3.79)	<0.01
Dietary fiber(g/1000kal)						
Q2：Q1					0.99(0.84-1.16)	0.872
Q3：Q1					0.82(0.69-0.98)	<0.05
Water intake						
<1500ml(female), <1700(male)					1(ref)	
≧1500ml(female),≧1700(male)					0.88(0.72-1.06)	0.173

PRAL: potential renal acid load; BMI: Body mass index; eGFR: Estimate glomerular filtration rate.

Table S2: Odds ratios (OR) and 95% confidence intervals for hyperuricemia by NEAP.

	**Crude model**	**P**	**model 2**	**P**	**model 3**	**P**
Age			0.97(0.97-0.98)	<0.01	0.97(0.96-0.98)	<0.01
Gender (Female: Male)			0.49(0.42-0.57)	<0.01	0.48(0.40-0.58)	<0.01
NEAP						

Q1	1(ref)	0.001	1(ref)		1(ref)	
Q2	1.39(1.15-1.67)	<0.001	1.25(1.03-1.53)	<0.05	1.19(0.97-1.45)	0.104
Q3	1.54(1.28-1.86)	<0.001	1.30(1.07-1.58)	<0.01	1.19(0.97-1.47)	0.1
Q4	1.69(1.40-2.02)	<0.001	1.40(1.15-1.70)	<0.01	1.25(1.00-1.56)	0.052
Marital status						
Married: Single			0.80(0.57-1.12)	0.185	0.84(0.60-1.18)	0.321
Other: Single			1.03(0.69-1.55)	0.882	1.07(0.71-1.61)	0.748
Region (Rural: Urban)			0.85(0.74-0.98)	0.025	0.86(0.74-0.99)	0.04
Education level						
Elementary school: None			0.90(0.73-1.11)	0.322	0.90(0.73-1.11)	0.313
Middle school: None			0.89(0.73-1.10)	0.28	0.89(0.73-1.10)	0.282
High school: None			0.96(0.74-1.23)	0.728	0.96(0.75-1.24)	0.769
Technical or vocational school: None			1.18(0.89-1.58)	0.256	1.19(0.89-1.59)	0.245
University or college: None			1.11(0.79-1.56)	0.555	1.12(0.79-1.57)	0.533
Smoking status (Yes: No)			0.96(0.81-1.14)	0.629	0.96(0.81-1.15)	0.667
Alcohol intake (Yes: No)			1.54(1.31-1.83)	<0.01	1.56(1.32-1.85)	<0.01
Hypertension (Yes: No)			1.43(1.24-1.66)	<0.01	1.44(1.24-1.67)	<0.01
Diabetes (Yes: No)			1.08(0.86-1.35)	0.523	1.08(0.86-1.35)	0.511
eGFR（ml/min/1.73m2）			0.95(0.94-0.95)	<0.01	0.95(0.94-0.95)	<0.01
BMI (kg/m2)						
<18.5: 18.5-24			0.57(0.39-0.84)	<0.01	0.57(0.39-0.84)	<0.01
24-28: 18.5-24			1.97(1.70-2.29)	<0.01	1.99(1.71-2.31)	<0.01
⩾28: 18.5-24			3.10(2.54-3.80)	<0.01	3.11(2.53-3.81)	<0.01
Dietary fiber(g/1000kal)						
Q2：Q1					0.96(0.81-1.14)	0.666
Q3：Q1					0.81(0.67-0.98)	<0.05
Water intake						
<1500ml(female), <1700(male)					1(ref)	
≧1500ml(female),≧1700(male)					0.89(0.73-1.07)	0.213

NEAP: net endogenous acid production; BMI: Body mass index; eGFR: Estimate glomerular filtration rate.

Table S3: Outcome of multiplicative interaction (PRAL).

Project	β	P	OR（95%CI）
**Dietary fiber & PRAL**			
Dietary fiber	-0.410	0.070	0.66(0.43-1.04)
PRAL	0.021	0.925	1.021(0.67-1.56)
Dietary fiber * PRAL	0.120	0.392	1.13(0.86-1.48)
Constant	4.578	0.000	
**Water intake & PRAL**			
Water intake	0.225	0.487	1.25(0.66-2.36)
PRAL	0.441	0.058	1.55(0.99-2.45)
Water intake * PRAL	-0.214	0.270	0.81(0.55-1.81)
Constant	3.901	0.000	49.470

PRAL: potential renal acid load; OR: Odds ratios; 95%CI: 95% confidence intervals.

Table S4: Outcome of multiplicative interaction (NEAP).

Project	β	P	OR（95%CI）
**Dietary fiber & NEAP**			
Dietary fiber	-0.479	0.039	0.62(0.39-0.98)
NEAP	-0.152	0.502	0.86(0.55-1.34)
Dietary fiber * NEAP	0.156	0.283	1.17(0.88-1.55)
Constant	4.903	0.000	134.69
**Water intake & NEAP**			
Water intake	-0.541	0.095	0.58（0.31-1.10）
NEAP	-0.235	0.315	0.79（0.50-1.25）
Water intake * NEAP	0.274	0.160	1.32（0.90-1.93）
Constant	5.036	0.000	153.814

NEAP: net endogenous acid production; OR: Odds ratios; 95%CI: 95% confidence intervals.

Table S5: Outcome of additive interraction(PRAL).

Measure	Estimate	Lower limit	Upper limit
**Dietary fiber & PRAL**			
RERI	0.079	-0.840	0.998
AP	0.104	-1.071	1.278
S	0.748	0.032	17.247
**Water intake & PRAL**			
RERI	-0.235	-2.291	1.820
AP	-0.150	-1.514	1.215
S	0.708	0.041	12.261

RERI：relative excess risk due to interaction; AP: attributable proportion due to interaction; S: the synergy index.

Table S6: Outcome of additive interraction(NEAP).

Measure	Estimate	Lower limit	Upper limit
**Dietary fiber & NEAP**			
RERI	0.143	-0.680	0.967
AP	0.231	-1.029	1.490
S	0.725	0.132	3.984
**Water intake & NEAP**			
RERI	0.233	-0.706	1.171
AP	0.384	-1.022	1.790
S	0.629	0.114	3.481

RERI：relative excess risk due to interaction; AP: attributable proportion due to interaction; S: the synergy index.
